# Coronavirus pandemic highlights critical gaps in rural Internet access for migrant and seasonal farmworkers: a call for partnership with medical libraries

**DOI:** 10.5195/jmla.2020.1045

**Published:** 2020-10-01

**Authors:** Joseph G. L. Lee, Catherine E. LePrevost, Emery L. Harwell, Jamie E. Bloss, Leslie E. Cofie, Melinda F. Wiggins, Gina C. Firnhaber

**Affiliations:** 1 leejose14@ecu.edu, Associate Professor, Department of Health Education and Promotion, East Carolina University, Greenville, NC; 2 celeprev@ncsu.edu, Teaching Associate Professor, Department of Applied Ecology, North Carolina State University and North Carolina Agromedicine Institute, Raleigh, NC; 3 elharwel@ncsu.edu, Research Assistant, Department of Applied Ecology, North Carolina State University, Raleigh, NC; 4 blossj19@ecu.edu, Assistant Professor, Laupus Health Sciences Library, East Carolina University, Greenville, NC; 5 cofiel18@ecu.edu, Assistant Professor, Department of Health Education and Promotion, East Carolina University, Greenville, NC; 6 melinda.wiggins@duke.edu, Executive Director, Student Action with Farmworkers, Durham, NC; 7 firnhaberg@ecu.edu, Assistant Professor, College of Nursing, East Carolina University, Greenville, NC

## Abstract

Migrant and seasonal farmworkers, who are essential workers in the coronavirus global public health emergency, face unique risks to their health as well as longstanding health inequities. This commentary highlights these risks and argues that Internet access represents an underappreciated but critical part of the public health response. The authors first discuss the unique risks farmworkers face. We note the importance of Internet access in the time of physical distancing, the fact that many health outreach workers are no longer visiting camps, the need for telemedicine infrastructure, and the role of Internet access in providing connections to families in communities of origin. We describe existing efforts that have been implemented in North Carolina to raise awareness among public health and health promotion practitioners and researchers. The current coronavirus pandemic demands the attention of medical libraries, public health practitioners, and policy makers to address the digital divide for farmworkers and their families.

The global public health emergency from severe acute respiratory syndrome coronavirus 2 (SARS-CoV-2), which we refer to as “coronavirus” for simplicity, has caused global disruptions and substantial morbidity and mortality. Many communities and states, including the authors' state of North Carolina (NC), have mandatory stay-at-home orders in place at the time of this writing. Essential workers, however, continue to be on the job, and, as Paul Farmer warned in *Infections and Inequalities: The Modern Plagues* [1], the consequences of existing structural inequalities are beginning to manifest in higher rates of infection and death for those with fewer resources [2].

One group of essential workers has unique vulnerabilities, which are created and maintained by having few legal protections: migrant and seasonal farmworkers [3, 4]. Migrant and seasonal farmworkers, who represent a critical part of rural economies and US food systems, face health inequities, poor and overcrowded housing conditions, limited access to personal protective equipment and handwashing facilities in the fields, and lack of access to health information [5, 6]. These issues required attention before the pandemic, and they are even more urgent now.

## PARTICULAR RISKS FOR MIGRANT AND SEASONAL FARMWORKERS

Farmworkers' living and working conditions may make it impossible for them to adhere to physical distancing guidelines, given existing minimum standards. Many workers, who arrive from Mexico on H2A Temporary Agricultural Workers visas or with a crew of migrant workers from another state in the United States, spend long hours together on a bus. Upon arrival, protections are limited: Beds in employer-provided migrant housing must be placed a minimum of three feet apart. In shared kitchens and bathrooms, one sink must be available for every six people, one stove for every ten, and one toilet for every fifteen [7].

These limited protections can make it difficult for farmworkers to comply with health recommendations. Indeed, the Farm Labor Organizing Committee (FLOC), a union of farmworkers, issued an emergency call on April 12, 2020, for donations of basic supplies such as cleaning products, handwashing soap, telephone cards, and toilet paper. FLOC's members reported arriving for the growing season to NC housing camps that lacked these critical supplies. Work conditions also involve risk. Depending on the crop and task, farmworkers work in large groups and in close proximity (e.g., riding on a tractor implement to transplant tobacco) in fields where the minimum washing facilities are set to be one per twenty people [8] but are sometimes altogether unavailable [9].

Connections to health care for farmworkers are limited by rural geography, language, and barriers to Internet access. Most farmworkers in NC live in isolated areas in rural, agricultural communities that have limited connectivity. There is longstanding evidence of inequities in access to the Internet by race, ethnicity, and rurality [10, 11], and these inequities extend to farmworkers [12–14].

Access to the Internet has been declared a basic human right by the United Nations in article 19 of the *Universal Declaration of Human Rights,* with economic and political reasons such as the rights to freedom of opinion and expression [15]. It is also important for accessing health information, connecting with health outreach workers during physical distancing, and maintaining connections with families who have remained in communities of origin.

The authors focus this commentary on the consequences of limited Internet access for farmworkers and their families during a pandemic and we note the need to substantially expand efforts that are underway to address this problem.

## TIMELY UPDATES TO INFORMATION ABOUT THE PANDEMIC

In a 2017–2018 pilot project with youth from seasonal farmworker families in central NC, half of participants did not have Internet access at home [16]. The importance of having Internet access is highlighted during this global pandemic, given that access to up-to-date information from reputable sources can mean the difference between stemming the spread of coronavirus or accelerating the rate of illness and death among groups who are most vulnerable [17].

During the coronavirus outbreak, there has been a wealth of information online for those who have Internet access. Yet, many farmworkers have no access to these important resources from state and local governments, health department websites, and worker organizations, and no way to keep abreast of new health information as it is discovered and disseminated. The quality of society's response to a global pandemic depends on meeting communication needs of all populations, with particular attention given to the most at-risk and vulnerable populations [18].

## DISCONNECTION FROM MIGRANT OUTREACH AND COMMUNITY HEALTH WORKERS

There are approximately 100 farmworker health outreach workers (i.e., community health workers or *promotoras de salud*) each agricultural season in NC. Outreach workers provide culturally sensitive and occupationally relevant health education, health assessments, and case management services, as well as organize access and transportation to clinical services [19]. Outreach is typically conducted in person as most employer-provided housing does not have phones or Internet.

To minimize risk to outreach workers as well as the spread between farmworker housing sites, many of NC's outreach workers are effectively grounded at the time of this writing. Some services can be administered virtually, such as sharing health information and organizing appointments with physicians or specialists, but outreach workers rely heavily on their ability to meet physically with farmworkers. In focus groups that were recently conducted with funding from the National Library of Medicine (award number G08LM013198), outreach workers in NC reported that in-person, interactive health education activities yielded the best results for engagement and information retention among farmworkers. Routine health assessments and clinic visits traditionally necessitate in-person interaction. The current pandemic and the resulting physical distancing requirements have removed a fundamental mode of service provision from outreach workers.

Turning to the existing virtual infrastructure, attempts by outreach workers to maintain connection with farmworkers are frustrated by limited and inconsistent access to Internet and cellular service for both farmworkers and outreach workers. Without regular site visits, many farmworkers' only point of connection to outreach workers is via phone or, if available, text messaging.

Under pandemic conditions, facilitation of telemedicine appointments and health information sharing are the only enabling services that outreach workers can administer. Telemedicine services require strong Internet connection and audiovisual hardware that, due to geography and economic conditions, are often unavailable to farmworkers. Even when the infrastructure does exist, online health information that is relevant to farmworkers is diffuse and not always culturally or linguistically sensitive. As outreach workers move to adapt service offerings and sustain connections with farmworkers in response to physical distancing requirements, the need for a more robust virtual infrastructure to support rural outreach work is clear.

## EMOTIONAL SUPPORT AND FAMILY CONNECTIONS

For many, Internet access provides now ubiquitous virtual meetings with friends, family, and coworkers in this time of physical distancing and stay-at-home orders. Previous work has shown that providing Internet access to migrant farmworkers allowed better connection with family members who remained in communities of origin [20]. Indeed, the most common applications (apps) used by farmworkers who were provided Internet in farmworker housing were social and communication apps like Facebook and messaging apps like WhatsApp [20]. These apps can serve as a means to gather and exchange vital information with loved ones. They are also a way for farmworkers to mobilize their social ties both in the United States and countries of origin to provide badly needed resources to their relatives and friends in the most vulnerable situations during a crisis. Without Internet access in this time of crisis, farmworkers are deprived of the ability to sustain connections to friends and families.

## EXISTING APPROACHES TO BRIDGING THE DIGITAL DIVIDE

The existing challenges caused by lack of Internet access have been recognized by researchers and advocates. Nonprofit organizations like Student Action with Farmworkers (SAF), based in Durham, NC, have pioneered early efforts to connect farmworkers with Internet access. Funded in part by the NC Farmworker Health Program, SAF's Computers and Connectivity project piloted efforts to place desktop computers and cellular WiFi hot spots in migrant housing at four labor camps across NC [20]. An external evaluator found many positive benefits for farmworkers in terms of emotional support from being able to connect with family [20].

In a separate project funded by the National Network of Libraries of Medicine, SAF partnered with East Carolina University's Department of Health Education and Promotion and Joyner Library to provide health information literacy training, iPads, and cellular data service to middle and high school students from farmworker families who were participating in a leadership development and college access program. The evaluation noted the substantial impact on the students of gaining Internet access for homework and access to health information [16].

## ONE MODEL FOR INCREASING INTERNET ACCESS AMONG MIGRANT AND SEASONAL FARMWORKERS

Our team is using funding from the National Library of Medicine (award number G08LM013198) to extend these two pilot projects with partnerships across NC using a train-the-trainer approach, development of best practices, identification of materials for wider use, and assessment of the sustainability of efforts to provide better access to the Internet. Our work will not, however, be able to fill the substantial gaps in Internet access for migrant and seasonal farmworkers in NC, much less nationally. Thus, it is critical to recognize the imperative prescribed by the coronavirus pandemic: Rural and farmworker health, medical libraries, emergency preparedness, education, rural economic development, and broadband infrastructure must be brought together to address barriers to Internet access in ways that include migrant and seasonal farmworkers.
